# FYVE domain-containing protein ZFYVE28 regulates EGFR-signaling in podocytes but is not critical for the function of filtration barrier in mice

**DOI:** 10.1038/s41598-018-23104-z

**Published:** 2018-03-16

**Authors:** Sonia Zambrano, Patricia Q. Rodriguez, Jing Guo, Katja Möller-Hackbarth, Angelina Schwarz, Jaakko Patrakka

**Affiliations:** KI/AZ Integrated CardioMetabolic Center, Department of Laboratory Medicine, Karolinska Institutet at Karolinska University Hospital Huddinge, Stockholm, Sweden

## Abstract

The kidney ultrafiltration barrier is formed of endothelial cells, the glomerular basement membrane and podocytes. Podocytes have a central role in normal physiology and disease pathogenesis of the glomerulus. Signaling through epidermal growth factor receptor (EGFR) in podocytes mediates development of many glomerular disease processes. In this work, we have identified zinc finger FYVE-type containing 28 (ZFYVE28) as a novel highly podocyte-enriched gene. We localize ZFYVE28 in podocyte foot processes in adult kidney. During glomerulogenesis, Zfyve28 is first expressed at the early capillary loop glomerulus. In cultured podocytes, we show that overexpression of ZFYVE28 promotes EGF-signaling, possibly by up-regulating EGFR expression and by modulating its localization. To study the role of ZFYVE28 *in vivo*, we generated both conventional and podocyte-specific knockout mouse lines. Kidneys developed normally in ZFYVE28-deficient mice. In adult mice, the absence of ZFYVE28 did not affect the maintenance of the filtration barrier. Moreover, ZFYVE28-deficiency did not affect the outcome of glomerular damage induced by injection of nephrotoxic serum. Taken together, we have identified Zfyve28 as a new molecular component of podocyte foot processes and show that it mediates EGF-signaling in podocytes. However, ZFYVE28 is not essential for the development or maintenance of the glomerulus filtration barrier.

## Introduction

The kidney filtration barrier located in the glomerulus is composed of three layers: (1) fenestrated endothelial cells, on the inside in contact with the blood; (2) the glomerular basement membrane (GBM), which serves as foundation and support; and (3) visceral epithelial cells (named podocytes), located on the outside of the glomerular capillary. The filtration barrier is responsible for sieving of plasma and production of primary urine, and its proper function is critical to prevent the leakage of plasma proteins into urine, a phenomenon known as proteinuria. Proteinuria is a hallmark sign of renal diseases.

Although all three layers forming the filtration barrier are necessary for its function, the podocyte seems to be the most critical constituent of the filter. This is highlighted for instance by the fact that mutations in many podocyte-specific proteins result in proteinuria^1,2^. Podocytes are terminally differentiated, post-mitotic cells with sophisticated structural features. They have prominent cell bodies with large cytoplasmic projections, named major processes, which further extend into smaller processes known as foot processes. The foot processes of adjacent podocytes are wrapping around the capillaries forming interdigitations. A highly specialized cell-cell junction termed the slit diaphragm connects adjacent foot processes^3^.

FYVE domain-containing proteins are a group of proteins involved in intracellular trafficking, signal transduction and regulation of the actin cytoskeleton^4–6^. Mammalian cells express more than 25 different FYVE domain-containing proteins. Their functions are still relatively poorly understood. Zinc finger FYVE-type containing 28 (ZFYVE28), also known as Lst2 protein, is a FYVE domain-protein that has been shown to be a regulator of the epidermal growth factor receptor (EGFR) pathway in both mammals^7^ and *C. elegans*^8^. In HELA cells, the non-ubiquitinylated form of ZFYVE28 co-localizes with EGFR in endosomes and promotes the degradation of the receptor. In the same article, they propose that ZFYVE28 could be involved in the recycling of EGFR to the membrane via its interaction together with the protein Trim3.

The activation of EGFR has been connected to the progression of several nephropathies like diabetic and membranous nephropathy, focal segmental glomerulosclerosis (FSGS) and rapidly progressive glomerulonephritis^9–12^. In knockout animals, deletion of EGFR specifically in podocytes protects mice from glomerular damage in diabetic nephropathy and rapidly progressive glomerulonephritis (RPGN). These studies demonstrate that EGFR activation has a major role in mediating podocyte damage in glomerular disease^9–11^.

In this work, we describe ZFYVE28 gene as a new podocyte-enriched gene that regulates EGFR signaling. Our studies in knockout animals indicate that ZFVYE28 is not essential for the development or maintenance of the glomerulus filtration barrier.

## Results

We have previously analyzed glomerular transcriptome through microarray profiling^13^. One of the glomerulus-enriched transcripts in that study encoded for Zfyve28 protein. As this protein has previously been linked to disease-associated EGFR signaling, we decided to characterize its expression and functional role in the glomerulus.

### ZFYVE28 mRNA is highly enriched in podocytes in both human and mouse

**Figure 1:**
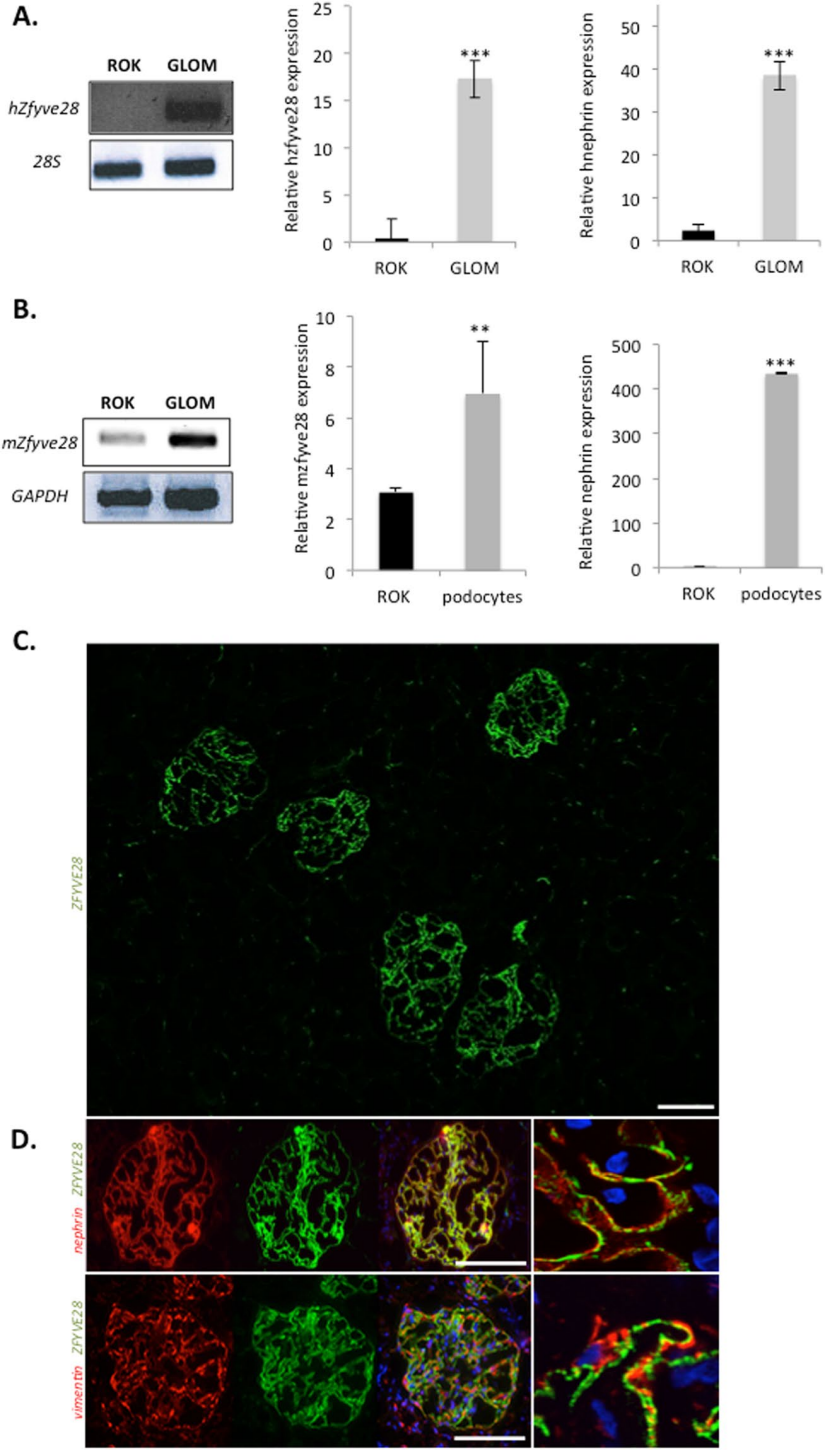
ZFYVE28 gene expression is enriched in podocytes. (**A**) In human kidney, ZFYVE8 transcript shows strong enrichment in the glomerulus (GLOM) when compared with the rest of the kidney (ROK) as detected by conventional RT-PCR (left) and qPCR (right). 28 S gene was used as a loading control and nephrin to validate the purity of glomerular fractions. Values are expressed as mean ± SD of 3 samples. ***p < 0.001. The pictures of the original gels are shown in the Supplementary Figure 1. (**B**) In mouse kidney, ZFYVE28 transcript is highly enriched in the glomerulus as detected by conventional RT-PCR (left). Quantitative PCR shows a strong enrichment of ZFYVE28 in isolated mouse podocytes. Nephrin was used to validate the purity of podocytes. GAPDH was used as a loading control. (**C**) Immunofluorescence staining for ZFYVE28 (green) in human kidney shows strong immunoreactivity in glomeruli and no significant signal in extra-glomerular areas. (**D**) Double staining with podocyte foot process marker nephrin (red) shows nearly complete co-localization (yellow). No significant overlapping reactivity was detected with podocyte major process marker vimentin (red). DAPI (blue) was used as a nucleus marker. Scale bars in C and D: 50 μm.

First, we wanted to validate experimentally the presence of ZFYVE28 transcript in the glomerulus. We studied the expression of ZFYVE28 in human kidney by comparing the isolated glomerular fraction to the tubulointerstitial fraction. The mRNA level of *Zfyve28* was significantly higher in the glomerular fraction as detected by RT-PCR and qPCR (Fig. 1A). By qPCR *Zfyve28* expression was 42-fold up-regulated in the glomerular fraction. The purity of human glomerular fractions was validated by measuring the expression level of nephrin (Fig. 1A).

In mouse kidney, *Zfyve28* transcript was in a similar fashion enriched in the glomerulus (Fig. 1B). To analyze which cell type in the glomerulus expressed ZFYVE28, we isolated mouse podocytes by generating a transgenic mouse line that expressed the tdTomato reporter specifically in podocytes. By using FACS, we were able to separate the podocyte fraction from rest of the kidney, as validated by nephrin expression (Fig. 1B). The qPCR analysis showed 6-fold up-regulation of *Zfyve28* mRNA in podocytes when compared to RNA gained from rest of the kidney (Fig. 1B). Taken together, these results indicate that ZFYVE28 mRNA expression is highly enriched podocytes.

### ZFYVE28 protein localizes to podocyte foot processes

Next, we analyzed the expression of ZFYVE28 at the protein level. Anti-ZFYVE28 antibody gave strong immunoreactivity in human glomeruli and only weak signal in extra-glomerular structures (Fig. 1C). Double labeling experiment with podocyte foot process marker nephrin showed almost complete overlapping of the two proteins in glomerular capillary loops (Fig. 1D). Double staining with vimentin, a marker of podocyte major processes, did not show any significant overlapping reactivity (Fig. 1D). Of note, the anti-ZFYVE28 antibody did not give any reliable signal in Western blotting experiments on isolated glomeruli (data not shown). Taken together, ZFYVE28 protein shows highly podocyte-enriched expression in the kidney and localizes to foot processes.

### During glomerulogenesis ZFYVE28 is first expressed in early capillary stage glomerulus

**Figure 2:**
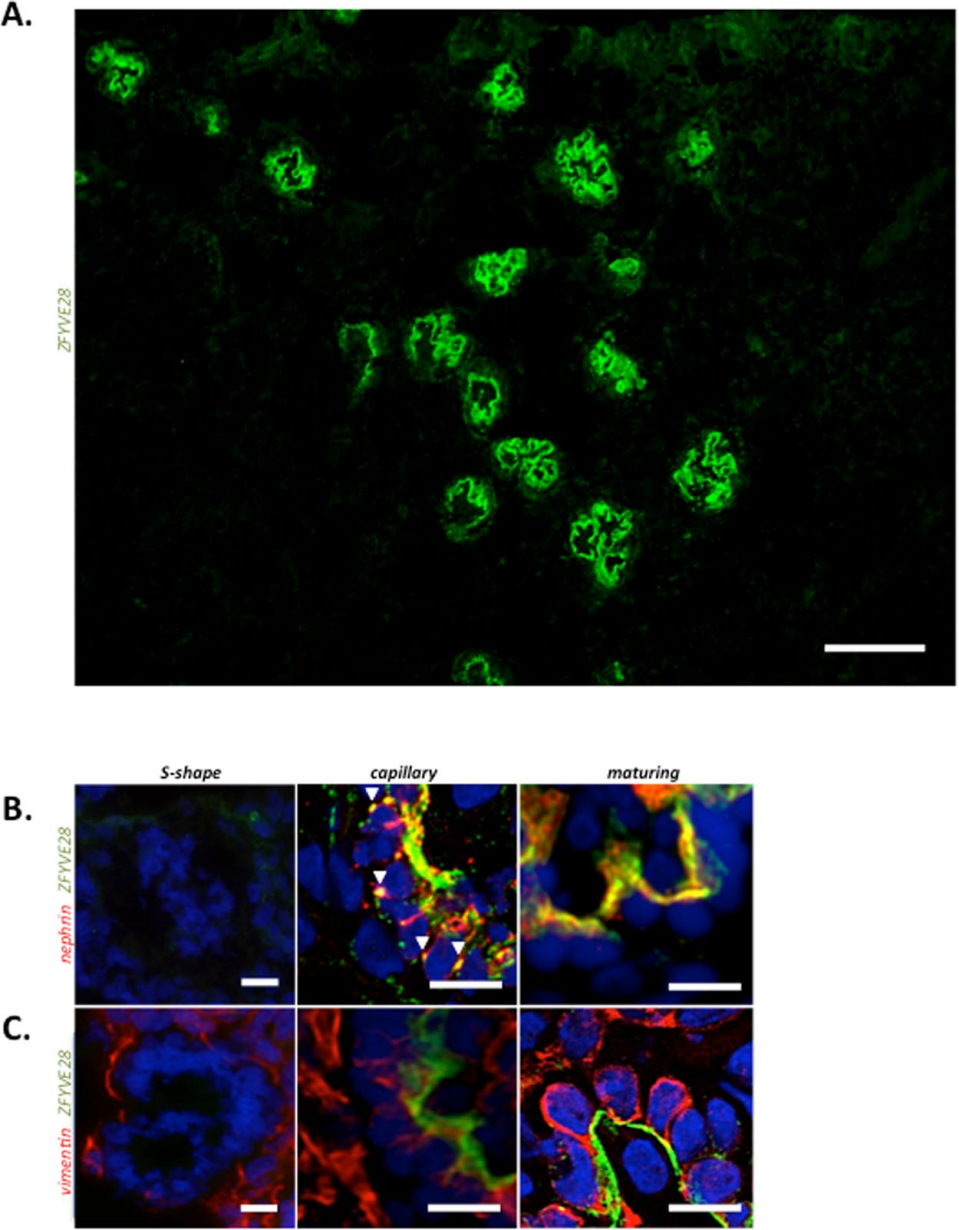
Expression of ZFYVE28 protein during human glomerulogenesis. **(A)** Immunofluorescence staining for ZFYVE28 (green) in human fetal kidney shows strong signal in developing glomeruli. Scale bar 50 μm. (**B**) S-shaped glomeruli show no staining for ZFYVE28 (green) or nephrin (red). At the capillary stage glomerulus ZFYVE28 co-localizes with nephrin at the basal aspects of pre-podocytes and between pre-podocytes (arrowheads). In maturing stage glomeruli, ZFYVE28 is detected at the basal side of developing podocytes. (**C**) Double labeling experiment with vimentin does not show co-localization during podocyte development. Scale bars 10 μm.

To validate the association of ZFYVE28 with podocytes, we analyzed its expression pattern during glomerulogenesis. Staining of human fetal kidney sections gave strong immunoreactivity in developing glomeruli (Fig. 2A). Earliest phases of glomerular development, vesicle and S-shaped glomeruli, showed no immunoreactivity for ZFYVE28 (Fig. 2B,C). ZFYVE28 was first detected at the early capillary stage glomerulus in where it was co-localizing with nephrin at the basal aspects of pre-podocytes (Fig. 2B). Interestingly, ZFYVE28 co-localized with nephrin also between pre-podocytes suggesting localization to developing slit diaphragms. In mature stage glomeruli, ZFYVE28 was detected at the basal side of developing podocytes. Double labeling experiments with major process marker vimentin did not show overlapping reactivity in developing glomeruli (Fig. 2C). Taken together, ZFYVE28 protein co-localizes with foot process markers during podocyte development supporting its association to these cellular structures.

### ZFYVE28 over-expression leads to up-regulation of EGFR expression and promotion of EGFR signaling in cultured podocytes

**Figure 3:**
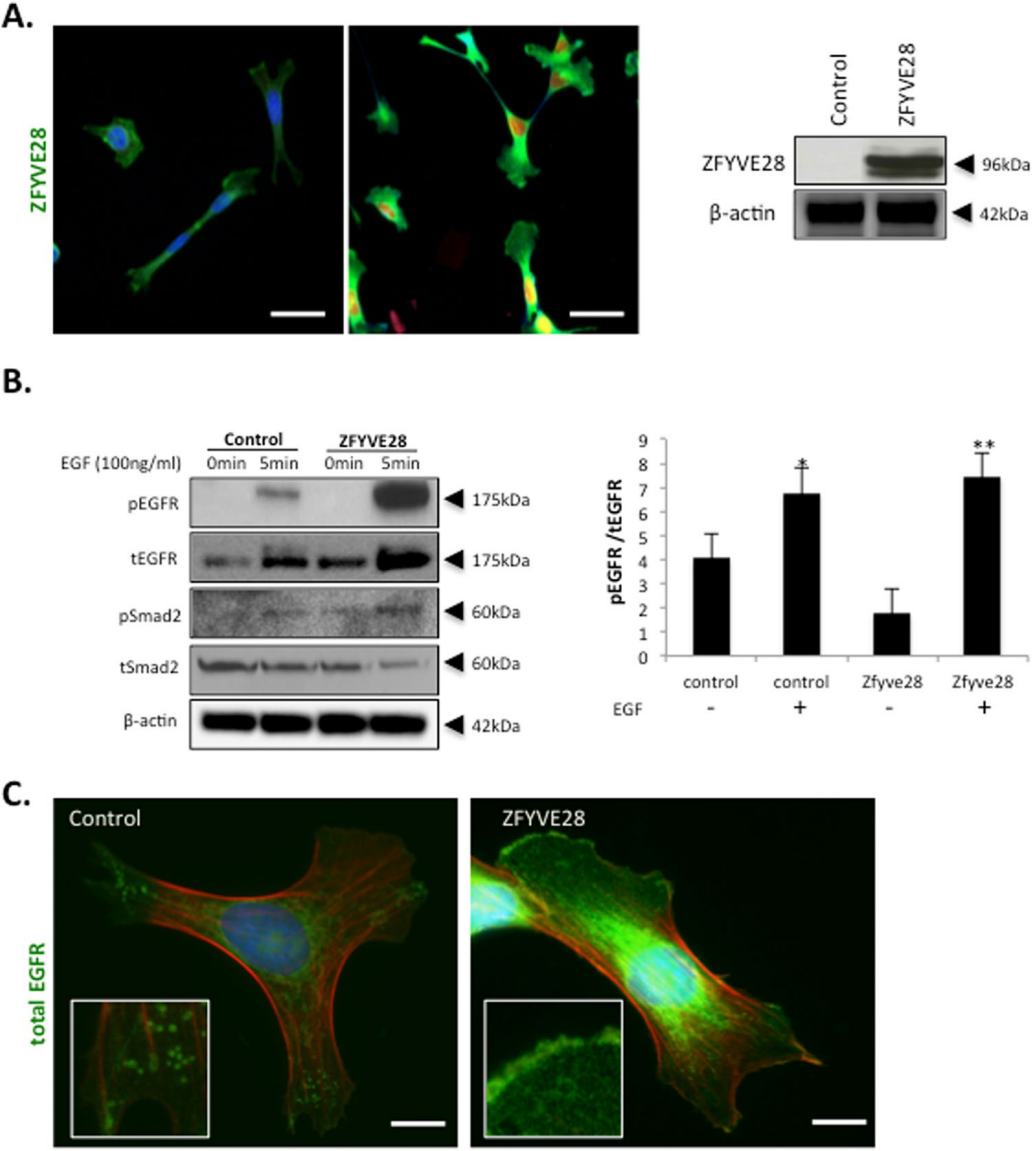
ZFYVE28 over-expression leads to up-regulation of EGFR expression in cultured human podocytes. (**A**) Immunofluorescence and Western blotting analysis of ZFYVE28 in control and over-expressing human podocytes. The western blotting belongs to the Clon 4 of cell over expressing Zfyve28, the complete blot with the rest of the clones appears in the Supplementary Figure 2. Staining is increased in over-expressing cells and the protein is detected in the cytosol of cells. Scale bar 10 μm. Western blotting shows a 96 kDa protein in transfected cells whereas control cells show no significant signal. Actin was used as a loading control. (**B**) Activation of EGFR, Smad2 is promoted in over-expressing cells after stimulation with EGF. The levels of total EGFR are also increased. Actin was used as a loading control. Densitometric quantification of the levels of phosphorylated and total EGFR shows similar ratios in both over-expressing and control cells. The full blots with the ladders appear in the Supplementary Figure 3. (**C**) Immunofluorescence analysis of EGFR in ZFYVE28 expressing cells shows increased staining and partial re-location to plasma membrane. Scale bar 5 μm.

We wanted to analyze the role of ZFYVE28 in podocyte biology using a well-established human podocyte cell line^14^. As cultured podocytes did not express ZFYVE28, we generated a stable ZFYVE28 over-expressing podocyte cell line. In the cell line, Zfyve28 protein was detected in the cytosol as detected by immunofluorescence staining (Fig. 3A). In Western blotting, anti-ZFyve28 antibody detected a 96-kDa protein in cell lysates, which is in line with published protein data^7^.

Previously, ZFYVE28 has been shown to act as a negative regulator of EGFR activity in HeLa cells^7^ and in C. Elegans^8^. Therefore, we analyzed this pathway in our podocyte cell line. Addition of EGFR ligand EGF induced the activation of the receptor as detected by increased phosphorylation (Fig. 3B). Importantly, the activation was clearly increased in cells overexpressing ZFYVE28. The stable over-expression induced a significant elevation in the total protein levels of EGFR (Fig. 3B). Phospho-EGFR/total EGFR ratios were similar in both overexpressing and control cells (Fig. 3B), suggesting that the increased EGFR phosphorylation after EGF treatment in ZFYVE28 cells was rather due to the up-regulation of total EGFR than promotion of receptor activation. As TGF-β signaling pathway has been shown to be downstream of EGFR activation in podocytes (9), we analyzed also this pathway. ZFYVE28 overexpression promoted the activation of as detected by increased phosphorylation (Fig. 3B).

Finally, we analyzed the expression of EGFR in cultured cells using immunofluorescence analysis. As detected by Western blotting, the presence of ZFYVE28 clearly increased the expression of EGFR (Fig. 3C). More interestingly, ZFYVE28 also seemed to partially re-locate EGFR in cells. In control cells, EGFR was found mostly in cytoplasmic vesicles, whereas in cells over-expressing ZFYVE28 the receptor was partially located at the plasma membrane (Fig. 3C).

### Generation of Zfyve28 knockout mouse lines

**Figure 4:**
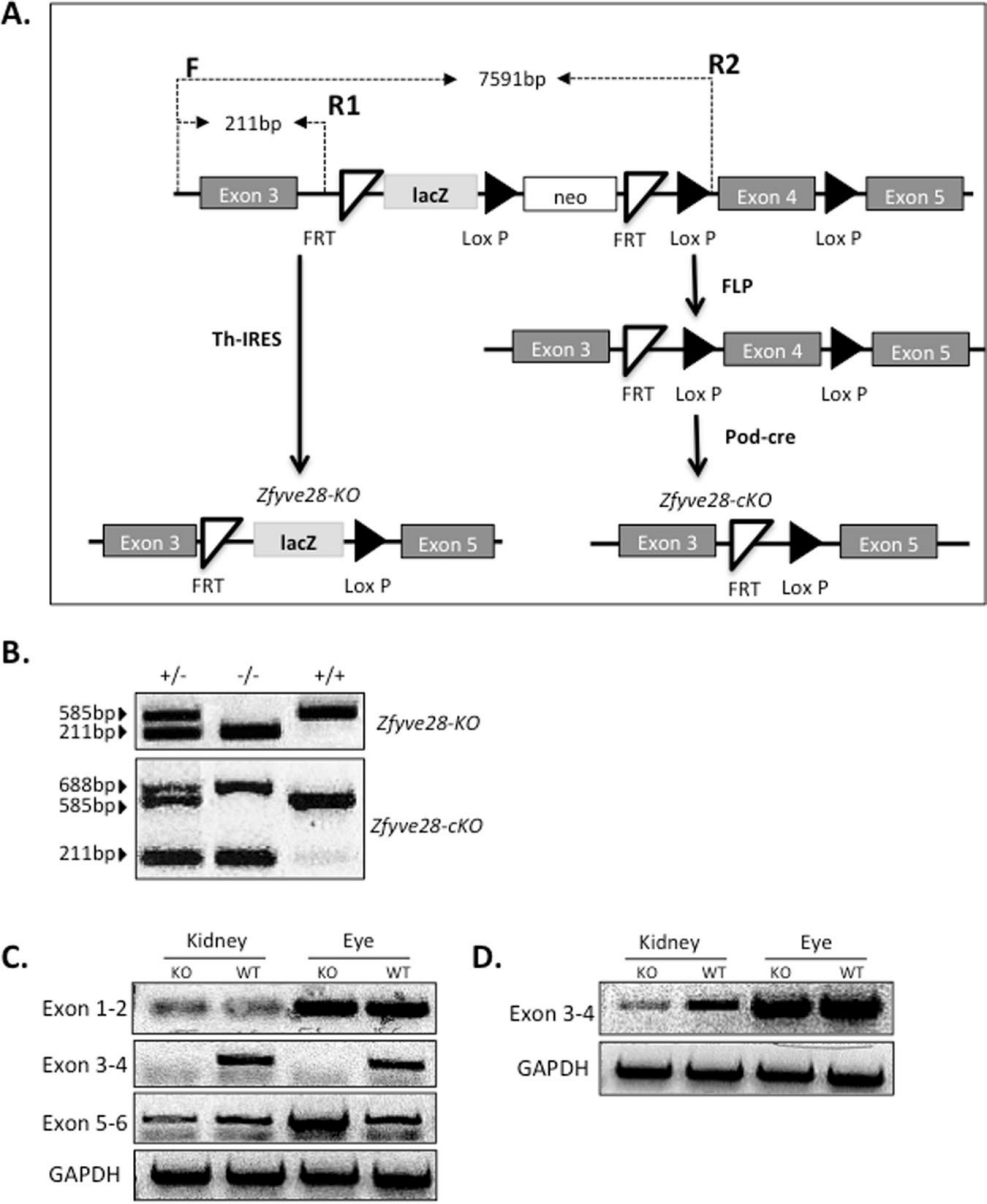
Generation of ZFYVE28 knockout mice. (**A**) Schematic drawing of the targeting strategy to generate full and conditional knockout mouse lines. The construct was targeted to surround the exon 4 of ZFYVE28 gene. The mice were crossed with Th-IRES deleter to generate a full knockout allele, and with FLP-deleter (FLP) line to generate a floxed mouse line. The floxed line was crossed with a podocin-cre line to inactivate ZFYVE28 specifically in podocytes. (**B**) In genotyping, full knockout allele generates a 211 bp PCR product, whereas wild type allele generates a 585 bp product. In floxed animals, an additional 688 bp product is detected. (**C**) In order to validate the deletion of the exon 4, we carried out PCR experiments using primes between exons 1 and 2, 3 and 4, as well as 5 and 6. In full KO animals, primer pair 3-4 cannot amplify a product in kidney and eye tissues, whereas in podocyte-specific KO animals, this pair can amplify a normal product in the eye but only a weak band is generated from kidney tissue. Full gels with ladder are shown in the Supplementary Figure 4.

To analyze the role of ZFYVE28 in the glomerulus *in vivo*, we generated a knockout (KO) mouse line for the ZFYVE28 gene. A transgenic mouse line in which the cassette containing lacZ and neomycin-resistance gene, as well as FRT and LoxP sites targeting the exon 4 of the ZFYVE28 gene was purchased from EUCOMM (www.eucomm.org) (Fig. 4A). We crossed the line with Th-IRES deleter line to generate a constitutive KO mouse line (Fig. 4A, referred as *Zfyve28*-KO). To generate a conditional KO allele, we crossed the transgene with a FLP-deleter line (Fig. 4A). In order to inactivate the gene in podocytes, we crossed this line with a Pod-cre line that express CRE under the podocin promoter (Fig. 4A). Podocyte-specific mice are referred as *Zfyve28*-cKO.

*Zfyve28*-KO and *Zfyve28*-cKO mice were genotyped by PCR. Wild type allele gave 585 bp fragment, whereas KO allele amplified a 211 bp product (Fig. 4B) those primers amplify an extra band of 688 bp in conditional knockout animals.

As anti-Zfyve28 antibody did not cross-react with the mouse protein (data not shown), we analyzed the transcript expression in detail to validate that the gene was successfully inactivated. We designed primer pairs to amplify sequences between exons 1–2, 3–4 and 5–6 of ZFYVE28 gene. In *Zfyve28*-KO mice, we could not amplify the sequence between exons 3–4 in eye or kidney tissue, indicating successful deletion of exon 4 (Fig. 4C). In *Zfyve28*-cKO animals, we could still detect a weak band for exons 3 and 4 in kidney tissue, whereas expression in the eye was unaffected (Fig. 4D). The remaining weak kidney expression is probably due to the incomplete inactivation of ZFYVE28 in podocytes or/and weak expression outside podocytes.

### Constitutive Zfyve28 knockout mice do not exhibit any kidney phenotype

**Figure 5:**
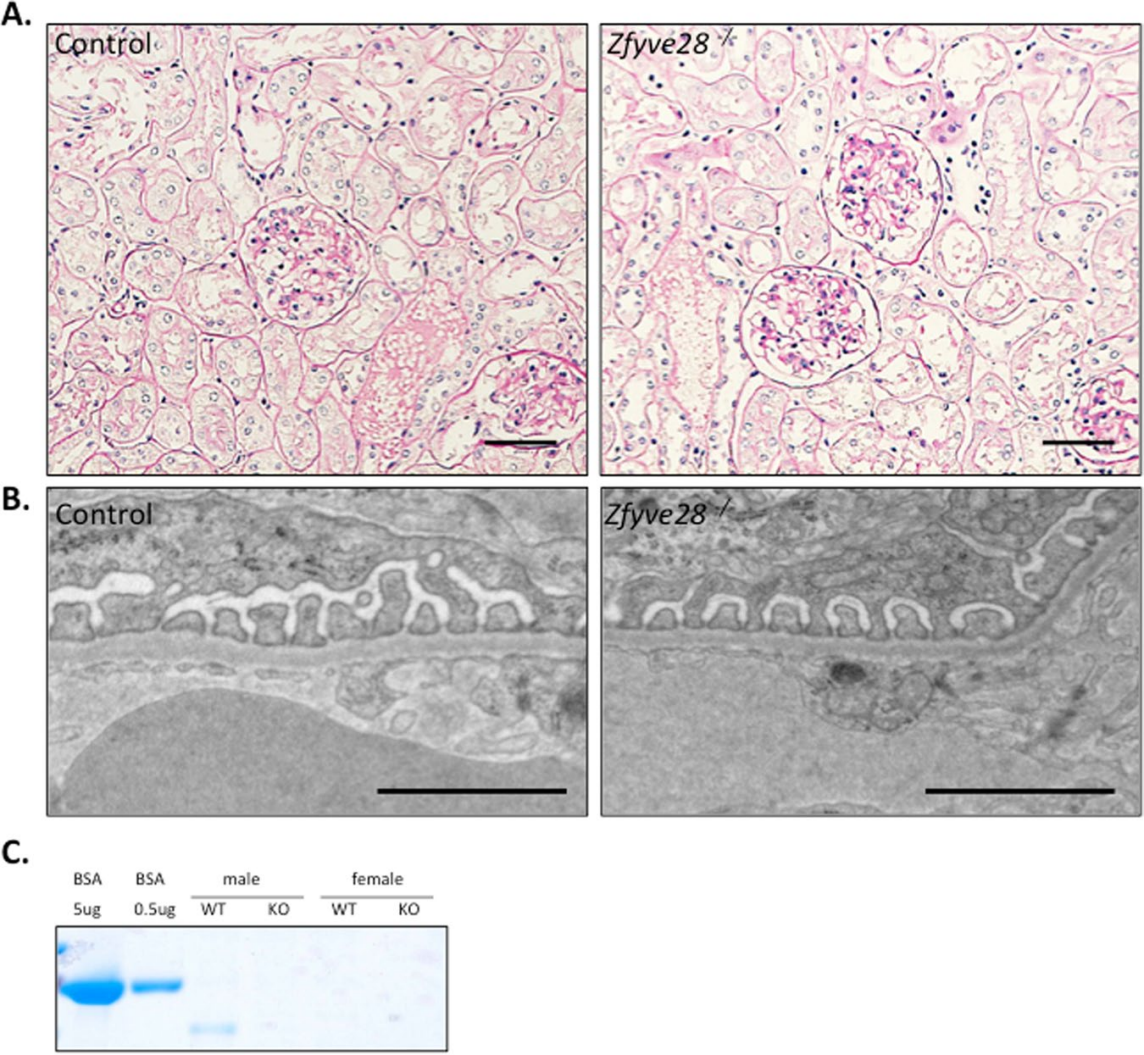
Constitutive ZFYVE28 knockout mice do not exhibit any obvious kidney phenotype. (**A**) PAS staining of kidneys in 6 months old KO mice shows normal glomerular and tubular morphology Scale bars 50 μm. (**B**) Electron microscopy shows intact glomerular filtration barrier in KO mice. Scale bar 250 nm. (**C**) No significant albuminuria is observed in urine analyses as detected by running 1 ul of urine to SDS-page gel (the full gel with ladder in the Supplementary Figure 5). BSA was used as a positive control.

*Zfyve28*-ko mice were born at an expected Mendelian ratio, had normal life-span and did not show any obvious abnormalities in major organs at macroscopic examination (data not shown). We examined the kidneys of 6 *Zfyve28*-ko and 6 littermate control mice to assess the importance of ZFYVE28 in the glomerulus. We could not detect any significant histological differences between KO and control mice in light microscopic examination at 6 months of age (Fig. 5A). Similarly, the ultrastructure of the filtration barrier remained intact after the deletion of *Zfyve28* as detected by electron microscopy (Fig. 5B). This included finely interdigitating podocyte foot processes interconnected by slit diaphragms. Similar analysis of *Zfyve28*-cKO animals did not show any renal abnormalities (data not shown). In order to analyze the renal function in KO animals, we measured the levels of albuminuria in mice at different time points during 1 year. No significant albuminuria was detected in urine analyses (Fig. 5C, data not shown).

### Absence of ZFYVE28 does not alter the response to EGFR-mediated anti-GBM glomerulonephritis

**Figure 6:**
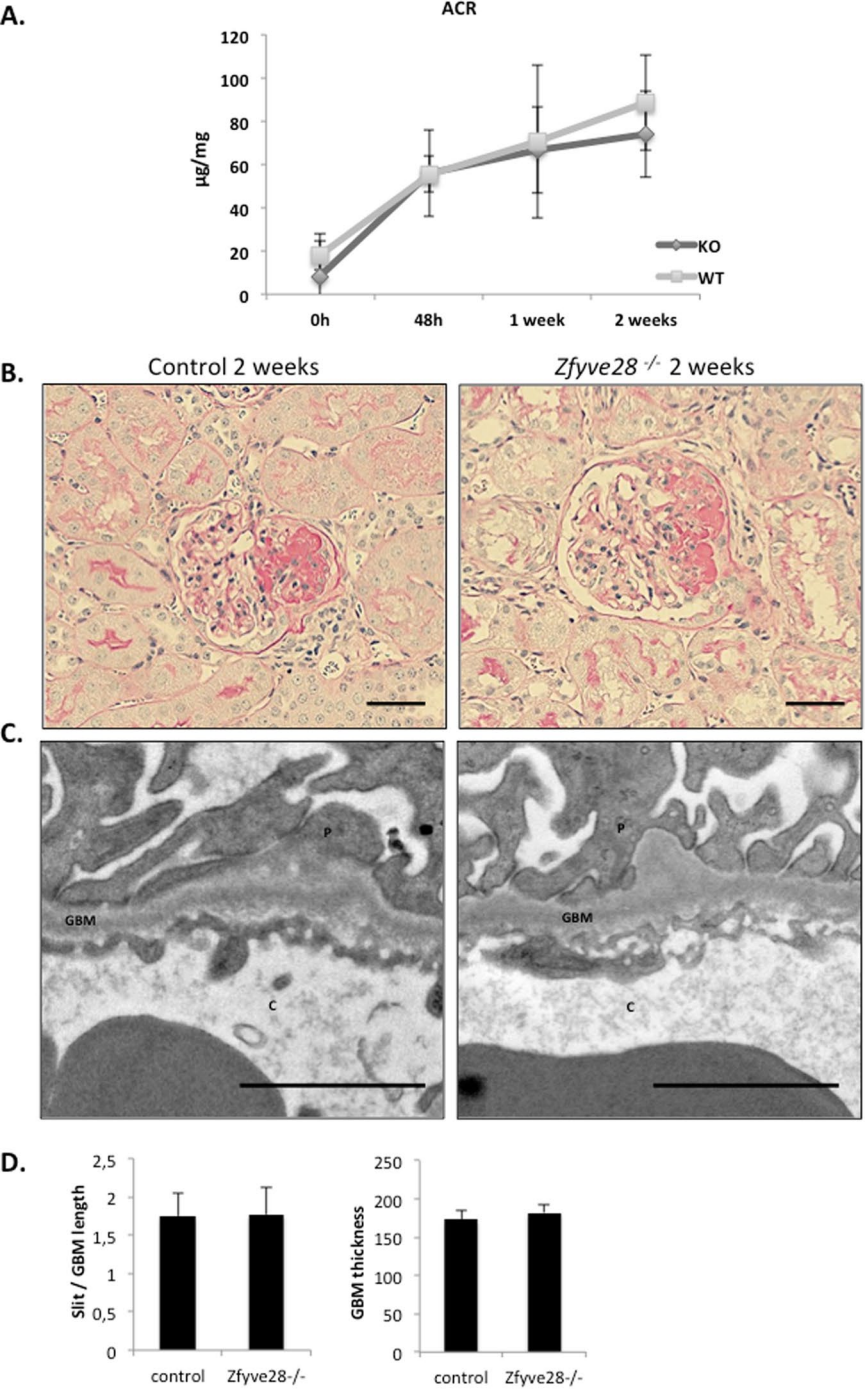
Absence of ZFYVE28 does not affect the outcome of anti-GBM glomerulonephritis. Anti-GBM glomerulonephritis was induced in 10 knockout and 10 littermate control animals. (**A**) Analysis of urine albumin/creatinine ratios shows that both KO and control mice develop albuminuria 48 h after the injection of anti-GBM serum. High albuminuria levels were maintained until the end of the experiment and no significant difference was observed between the groups. (**B**) PAS staining of kidneys 2 weeks after the induction shows segmental sclerotic changes in both KO and control mice. (**C**) Electron microscopic examination shows foot process effacement in both KO and control mice. Scale bar 250 nm. (**D**) The quantification of foot process effacement shows no difference between KO and control animals. The thickness of the GBM is also similar in both groups.

The pathogenic role of EGFR in podocytes has previously been shown in an anti-GBM glomerulonephritis model (anti-GMB GN)^9,10^. Therefore, we decided to induce this disease in Zfyve28-deficient mice. In the model, a single injection of anti-GBM anti-serum causes severe glomerular damage and albuminuria^15^. In our experiment, both KO and control mice showed clear albuminuria 48 h after the injection of anti-serum (Fig. 6A). High albuminuria levels were maintained until the end of the experiment (2 weeks later). However, no significant differences were detected in albuminuria levels between KO and control mice. Histologically, most animals injected with anti-serum presented focal segmental glomerulosclerosis (Fig. 6B, Table 1). Occasionally, totally sclerotic glomeruli and proteinaceous casts in tubuli were detected. We measured the level of histological progression of the disease by analyzing the number of sclerotic glomerular changes and the presence of casts, and could not observe any significant difference between KO and control animals (Table 1). The EM analysis revealed partial foot processes effacement, but no significant differences between KO and control animals were observed (control animals 137 ± 4, knockout animals 143 ± 18) (Fig. 6C).

**Table 1:** Histological changes in Zfyve28 knockout and control animals 2 weeks after induction of anti-GBM glomerulonephritis. Data shown as media ± standard deviation.

Genotype	Global histology		
	Normal	Segmental sclerosis	Global sclerosis
Control	89.2 ± 3.5	7.6 ± 2.0 (5–11)	3.0 ± 1.3 (2–5)
Knockout	90.2 ± 4.8 (85–100)	7.3 ± 3.7 (0–11)	2.8 ± 1.6 (0–5)

## Discussion

The podocyte plays a key role in the physiology and diseases of the glomerular filtration barrier^16–18^. Several signaling pathways, such as EGF, have been pinpointed to drive the progression of kidney disease in podocytes. Therefore, it is important to understand mechanisms regulating podocyte biology in health and disease. In this study, we show that: 1) The FYVE domain-containing protein ZFYVE28 is highly enriched in both human and mouse podocytes wherein it localizes to foot processes; 2) ZFYVE28 promotes EGFR-mediated signaling in cultured podocytes; 3) *In vivo*, ZFYVE28 is not needed for the development or maintenance of the glomerular filtration barrier as ZFYVE28-deficient mice do not exhibit any obvious renal phenotype.

Originally, we identified ZFYVE28 by a microarray-based study in which the gene was enriched in the mouse glomerulus transcriptome. We validated this mRNA enrichment using RT-PCR and qPCR. Furthermore, by FAC-sorting podocytes from a transgenic mouse line, we could show that it was specifically expressed by podocytes in the glomerulus. We confirmed this at the protein level by detecting predominant podocyte staining in human kidney sections, and moreover, localized the protein to podocyte foot processes. Although this antibody did not give a reliable signal in Western blotting experiments or in mouse tissues, we conclude that our detailed expressional profiling, both at the mRNA and protein level, indicates that *Zfyve28* is a novel highly podocyte-enriched gene.

To clarify its role in podocytes, we analyzed ZFYVE28 in an established human podocyte cell line^14^. As the cell line does not show significant endogenous ZFYVE28 expression, we generated a stable overexpressing cell line. ZFYVE28 has been reported to associate with EGFR signaling pathway^7,8^. As EGFR is involved in the development of several nephropathies^9,11,12^, we analyzed this signaling pathway in our cell culture model. Interestingly, the levels of total EGFR were significantly elevated in cells that over-expressed ZFYVE28. This promoted the activation (phosphorylation) of EGFR after EGF exposure, and phosphorylation of Smad2, a downstream target of EGFR signaling (Fig. 3 and Supplementary Figure 6). In addition, the over-expression of ZFYVE28 seemed to affect the location of EGFR, as the receptor was located predominantly at the cell surface in this cell line. This could explain, at least in part, why these cells are more sensitive to the treatment with EGF. In a work published by Mosesson *et al*.^7^, they show how ZFYVE28 is playing a role in the location and fate of EGFR via mono-ubiquitinylation and phosphorylation in HeLa cells. Here we show, for first time, that ZFYVE28 seems to act as a positive modulator of the EGFR pathway in podocytes.

We localized ZFYVE28 to the foot processes of podocytes. During glomerulogenesis ZFYVE28 co-localized with nephrin between developing podocytes, suggesting association to the slit diaphragm. The slit diaphragm is critical for the filtration barrier and mutations in slit diaphragm-specific proteins cause inherited forms of proteinuria^2^. To understand the role of ZFYVE28 in podocytes, we generated constitutive and podocyte-specific *Zfyve28* KO mouse lines. The *Zfyve28*-KO animals did not present any abnormalities in renal function or in the structure of the ultrafiltration barrier indicating that ZFYVE28 is not essential for the development and function of the glomerulus filtration barrier.

Based on our results *in vitro*, we decided to challenge *Zfyve28* KO mice with a model of RPGN, as EGFR activation in podocytes has been shown to drive the disease process^9,10^. However, the ZFYVE28-deficient animals developed renal disease in a very similar way as the controls in this experimental setting. There were no differences between the groups as detected by measurement of albuminuria and BUN, or by histological examination. Thus, our analyses did not reveal a potential functional role of ZFYVE28 in response to glomerular injury, at least within the model and time lines evaluated in this study. This could be due to different factors. For example: a genetic redundancy may exist for the function that ZFYVE28 performs in podocytes. Alternatively, it could be due to compensatory mechanisms, for instance the EGFR trafficking is complex and can be carried out by a variety of molecules and biological processes^19–21^.

In conclusion, we describe ZFYVE28 as a novel podocyte-enriched protein that seems to be a positive modulator of EGFR signaling in podocytes. Studies in ZFYVE28-deficient mice indicate that it is not necessary for the development or maintenance of the normal glomerulus filtration barrier.

## Materials and Methods

### Human material

Human renal tissue was collected from kidneys surgically removed because of renal cell carcinoma. Human fetal kidney samples were collected from a 20-week old fetus, nonviable due to neural tube defects and hydrocephalus (University Hospital of Helsinki, Finland). The collection of material was approved by the local ethical committee (University Hospital of Helsinki, Finland). All experiments were performed in accordance with relevant guidelines and regulations.

### Transgenic mouse lines

Gene targeting of ZFYVE28 gene was performed by EUCOMM (www.eucomm.org). The construct was targeted to surround the exon 4 of ZFYVE28 gene. The mice were in a mixed C57vl/6 and 129 Sv background. We crossed these mice with Th-IRES deletor to generate a full knockout allele, as well as with an FLP-deleter line to generate a floxed mouse line flox *Zfyve28* (*Zfyve28-fl*). *Zfyve28-fl* was crossed with a podocin-cre line to inactivate *Zfyve28* specifically in podocytes (*Zfyve28*-cKO). The genotyping was done by PCR using genomic DNA extracted from ear biopsies. Primers for genotyping were: CAS-R1-Term: tgctggtatcgttatgcgcc, *Zfyve28*-48507-F: cgctccccttcaggatacac, *Zfyve28*-48506-R: tcatcatggcctcagtgctc. The inactivation of the gene was further validated by isolating total RNA from kidneys and by performing PCR using primers designed to amplify the area of the transcript corresponding to the region between the exons 1–2, 3–4 and 5–6 of the ZFYVE28 gene. The primers used were: exon1-2-F: tggttcagctcctcatcagc, exon1-2-R: aacccaagaggtcagaccca, exon3-4-F: aggagatccggcatgacaac, exon3-4-R: aggacatcaaagcgcctcag, exon5-6-F: cactttcagccagtgccaac and exon5-6-R: gtgttccctgggcattcagg.

The Gt(ROSA)26Sor^tm14(CAG-tdTomato)Hze/J^ mice were kindly provided by Daniel Nyqvist (Department of Medical Biochemistry and Biophysics, Karolinska Institute), and were crossed with a podocin-cre line to activate the tdTomato expression specifically in podocytes. Breeding and genotyping was done according to standard procedures. All animal studies were carried out in Scheelelaboratoriet (Karolinska Institutet) and were approved by the ethical Committee on Research Animal Care (Stockholms Norra djurförsöksestiska nämd). All experiments were performed in accordance with relevant guidelines and regulations.

### Podocytes in cell culture

Human podocytes were cultured as previously described^14^. Briefly, podocytes were grown under permissive conditions at 33 °C temperature in media containing 0.01 mg/ml recombinant human insulin, 0.0055 mg/ml human transferrin. We developed stable podocyte cell lines expressing ZFYVE28 by transfecting the cells using Lipofectamine 2000 (Thermo fisher scientific). The cells were transfected with pRP(Exp)-CMV > hZFVE28(ORF0226721):T2A:Puro/3xFLAG (VectorBoulder.com) or with an empty vector. Clones were obtained by limited dilution and expanded under puromycin selection. The over-expression of ZFYVE28 in different clones was confirmed by western blotting. The cells were treated with 100 ng/ml of recombinant human HB-EGF (R&D systems, cat: 259-HE) at different time points.

### Immunofluorescence

We used frozen human samples that were cut in slices of 5 μm. The tissue sections were fixed during 20 min in cold acetone and blocked for 1 h with normal goat serum. The primary antibodies anti-ZFYVE28 (Atlas antibodies, cat: HPA038175) and anti-total-EGFR (Abcam, cat: ab2430) were incubated over night at 4 °C (dilutions 1:500 and 1:1000 respectively). The nephrin antibody has been described previously^22^ and the vimentin antibody was purchased for Sigma (cat: V5255) Those two antibodies were incubated for 1 h at room temperature (Dilution 1:1000).

### Histological analysis

For histological analyses, kidney samples were fixed in 4% paraformaldehyde followed by dehydration and embedding in paraffin. At least 8 control and 8 KO animals were analyzed in each experiment. HE and PAS staining were done using standard protocol. Transmission electron microscopy was performed on renal cortex fixed with glutaraldehyde following standard procedures.

### Quantitative and conventional PCR

Glomeruli were isolated from human and mouse kidneys as published previously^13,23^. All the qPCRs were performed following the standard methods and using SYBR Green (Biorad, cat: 1725271) and the conventional PCR were performed using HotStarTaq polymerase (Quiagen, cat: 03643). The used primers were: hu*Zfyve28*-F: cggaaaataagggatttgct, hu*Zfyve28*-R: cacgtcatccatgaagaaca, mm*Zfyve28*-F: tcctgtgtgtgctgtgggag, mm*Zfyve28*-R: gggctctttgtcaccaccct, hu28S-F: ttgaaaatccgggggagag, hu28S-R: acattgttccaacatgccag, mm*Uchl*-1-F: gtcatctacccgacactggc, mm*Uchl*-1-R: gtcatctacccgacactggc, mmGAPDH-F: tgttcctacccccaatgtgt, mmGAPDH-R: tgtgagggagatgctcagtg.

### Western blotting

Western blotting was performed using standard procedures. Antibodies anti-Zfyve28 (Human protein atlas. Dilution 1:500), anti-pSmad2 (Cell signaling, cat: D27F4. Dilution 1:1000), anti-total-EGFR (Abcam, cat: ab2430. Dilution 1:250) and anti-active-EGFR (Cell signaling, cat: 2234. Dilution 1:1000) were incubated ON at 4 °C. For detection method, we used Clarity western ECL substrate (BioRad, cat: 1705061) and the ChemiDoc touch imaging system (BioRad).

### Urine and plasma analysis

Urine samples were collected from mice, and the presence of albuminuria was initially analyzed by running 2 µl of urine on SDS-PAGE gel stained with PAGE-Blue stain. Then, we calculate the albumin/creatinin ratio by using the commercial kits Albuwell (Exocell, cat: 1011) and Quantichrome creatinine assay kit (Bio assay systems, cat: DICT-500). The blood was collected by cardiac puncture and added immediately into a tube containing a serum separator additive (BD Microtainer, cat: 365967) and centrifuged 10 min at 1100 rcf. The plasma samples were sent to the *Universitetsdjursjukhuset*, Uppsala (Sweden) to carry out the analysis of different parameters. All experiments were performed in accordance with relevant guidelines and regulations.

### Anti-GBM glomerulonephritis model

For this study 20 animals (10 knockout and 10 wild type) 8 week old, were pre-immunized subcutaneously with 100 µl of sheep IgG Freund´s complete adjuvant (Sigma, cat: F5881). Four days later, glomerulonephritis was induced by intravenous injection of 100 µl of anti-GBM serum purchased from Probetex (cat: PTX-001S). Experimental mice were maintained under standard animal house conditions in a conventional animal facility during 14 days. After that period, the animals were anaesthetized by intraperitoneal injection of avertin (200 mg/kg) in order to proceed to the organ collection. All animal studies were carried out in Scheelelaboratoriet (Karolinska Institutet) and were approved by the ethical Committee on Research Animal Care (Stockholms Norra djurförsöksestiska nämd). All experiments were performed in accordance with relevant guidelines and regulations.

### Isolation of mouse glomeruli

The mouse glomeruli were isolated using magnetic beads method, described by Takemoto *et al*.^13^.

### Podocyte isolation

The isolated glomeruli were re-suspended in 2 ml of digestion buffer (collagenase V 1 mg/ml, pronase E 1 mg/ml and DNase I 50 U/ml) and incubated for 45 min at 37 °C on a thermomixer shaking at 1400 rpm. After the incubation the suspension was sheared with an insulin needle (29 G). We used the particle concentrator in order to remove the magnetic beads remaining. Then, we passed the suspension through a pre-wet sieve (30 μm), and collected the cells by centrifugation at 1500 rpm for 5 min at 4 °C. The cells were re-suspended in 400 µl of sterile HBSS (2 kidneys in 400 μl of HBSS). To separate the tdTomato positive cells, the cell suspension was sorted with a FACS sorter Aria III sorter. We detected tdTomato by using a 561 nm laser and 585/15 filter. Around 100000 podocytes were obtained per mouse.

### Statistic

Statistical difference between two groups was analyzed by t-test. Data was analyzed using the GraphPad. Prism software (San Diego, CA) and was recorded as the mean ± standard error with p > 0.05 defined as significant.

## Electronic supplementary material

Supplementary figures
