# Supplementary material for: FYVE domain-containing protein ZFYVE28 regulates EGFR-signaling in podocytes but is not critical for the function of filtration barrier in mice
Source: Sci Rep. 2018 Mar 16;8:4712. doi: 10.1038/s41598-018-23104-z (PMC5856817; doi:10.1038/s41598-018-23104-z)
Supplement: Supplementary file 1 — Supplementary figures [file 41598_2018_23104_MOESM1_ESM.pdf]

Supplementary material for

FYVE domain-containing protein ZFYVE28 regulates EGFR-signaling in podocytes but is not critical for the function of filtration barrier in mice

Sonia Zambrano Sevilla[1], Patricia Q Rodriguez[1], Jing Guo[1], Katja Möller-Hackbarth[1], Angelina Schwarz [1], Jaakko Patrakka[1]

[1]KI/AZ Integrated CardioMetabolic Center, Department of Laboratory Medicine, Karolinska Institutet at Karolinska University Hospital Huddinge, Stockholm, Sweden

Supplement figure 1

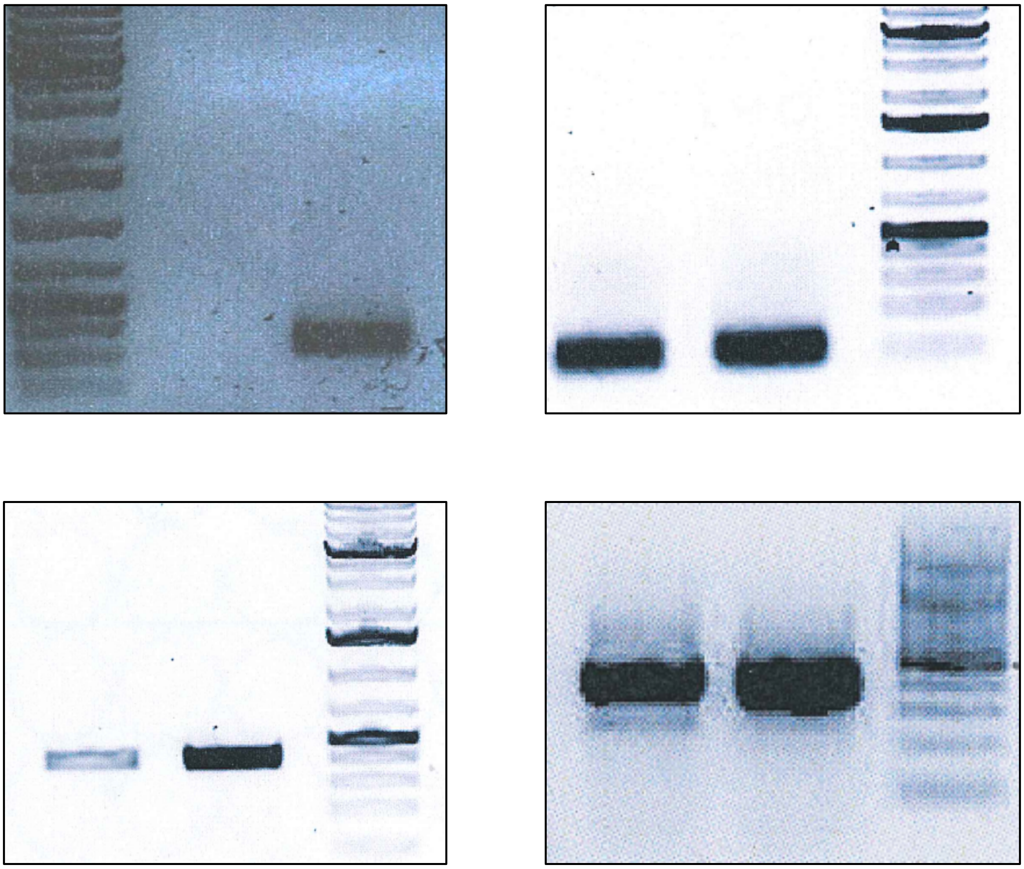

Supplementary figure 1. Full image of the gels from figure 4. The marks correspond to the GeneRuler 100bp DNA Ladder, Thermo Fisher.

Supplement figure 2

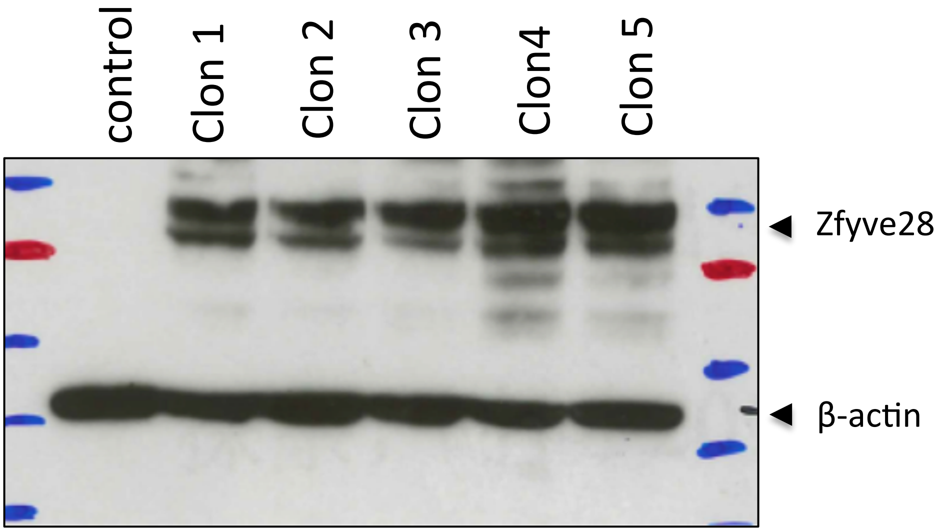

Supplementary figure 2. Western blotting analysis of ZFYVE28 in control and over-expressing human podocytes clones. The marks correspond to the PageRuler prestained protein ladder 10-170 KDa (Fermentas)

Supplement figure 3

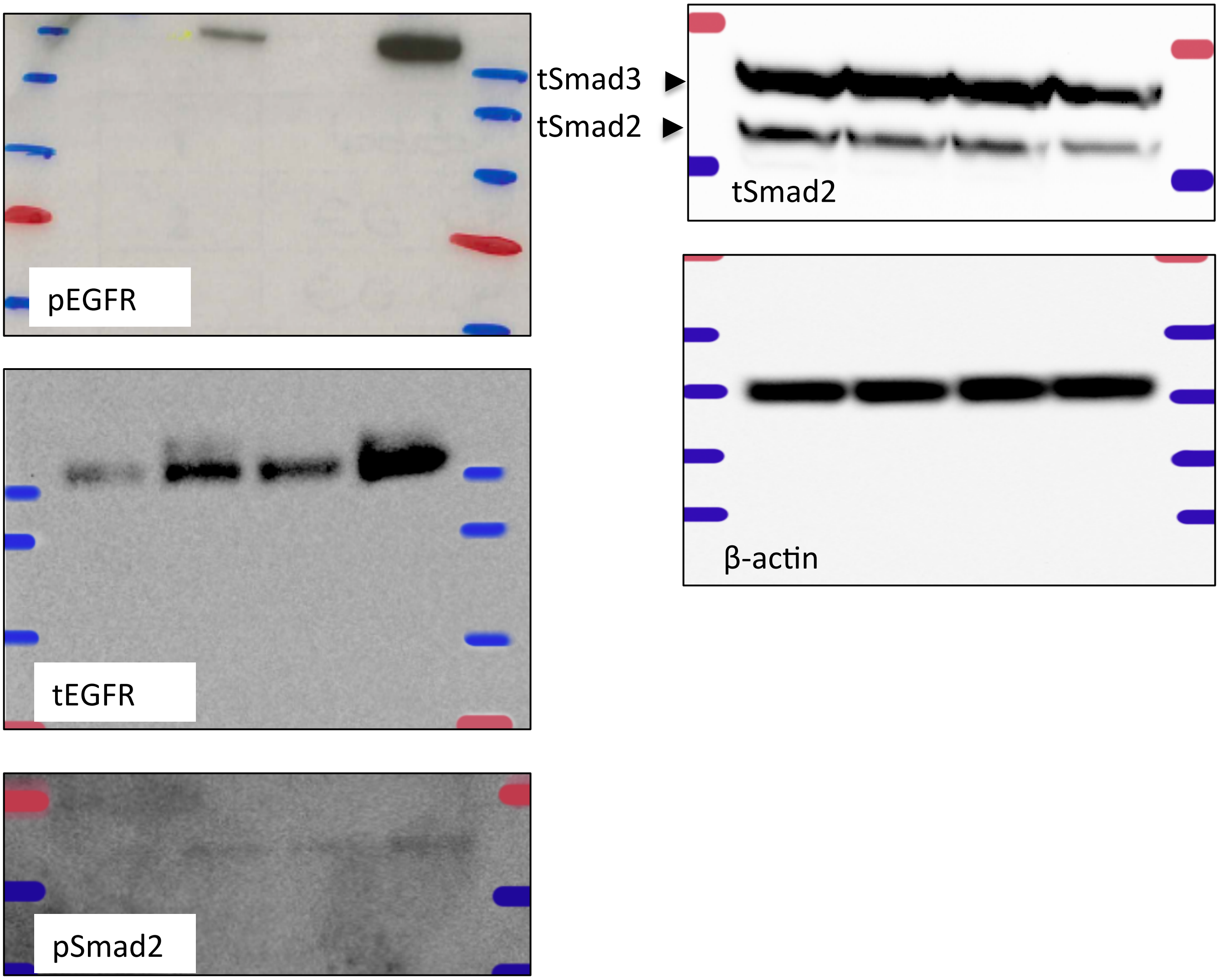

Supplementary figure 3. full image of the blots from figure 3. The marks correspond to the PageRuler prestained protein ladder 10-170 KDa (Fermentas)

Supplement figure 4

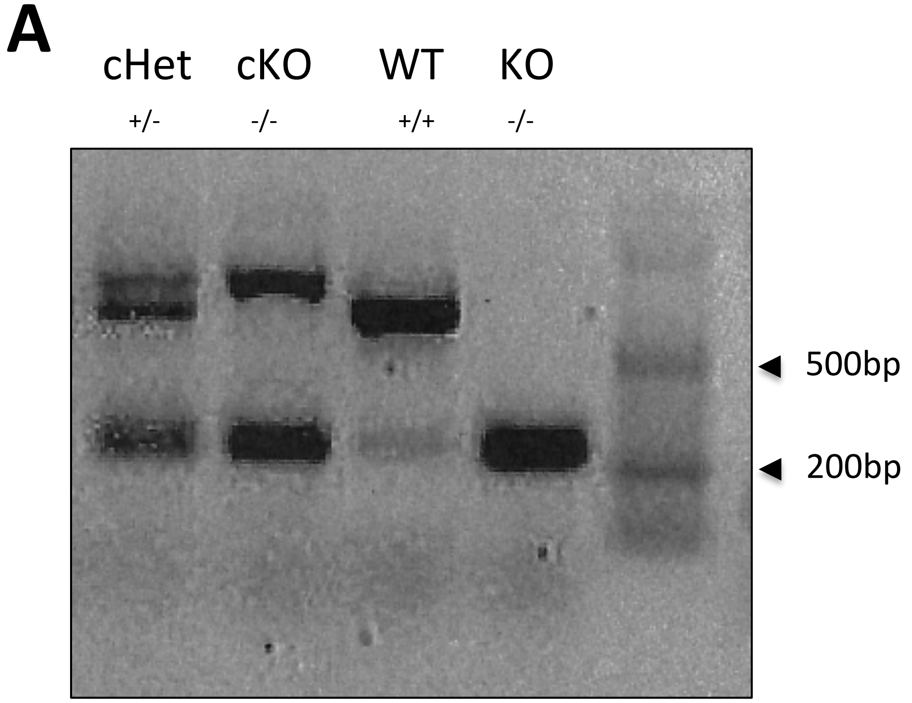

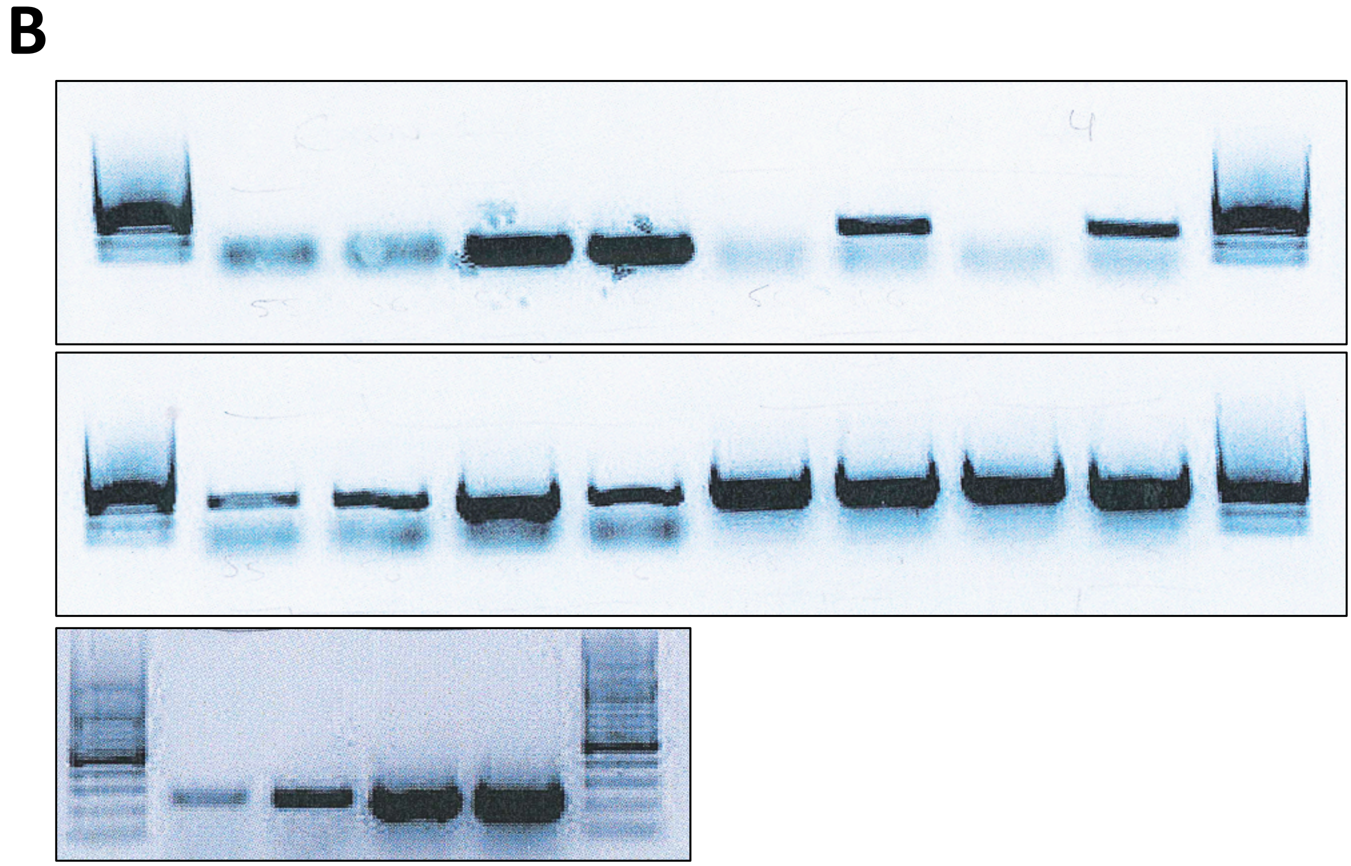

Supplementary figure 4. (A-B) Full image of the gels from figure 4. The marks correspond to the GeneRuler 50bp DNA Ladder, Thermo Fisher.

Supplement figure 5

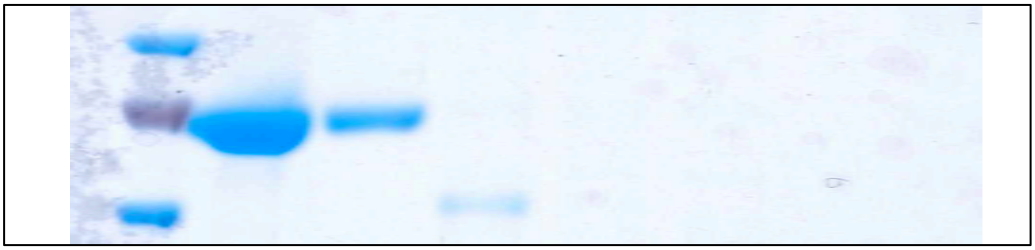

Supplementary figure 5. full image of the grl from figure 5. The marks correspond to the PageRuler prestained protein ladder 10-170 KDa (Fermentas)

Supplement figure 6

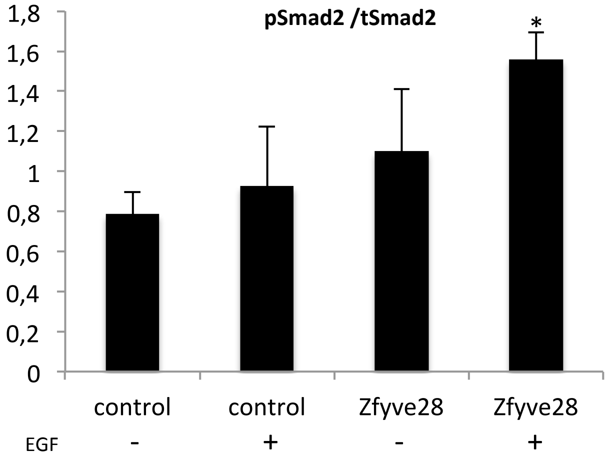

Supplementary figure 6. Ratio pSmad2/tSmad2. The standard error has been calculated from 3 independent experiment.
